# Estimation of skeletal muscle mass in 4-year-old children using the D_3_-creatine dilution method

**DOI:** 10.1038/s41390-023-02587-1

**Published:** 2023-04-10

**Authors:** Aysha Sidiqi, Farzana Fariha, Shaila S. Shanta, Alison Dasiewicz, Abdullah Al. Mahmud, Daniel R. Moore, Mahalakshmi Shankaran, Marc K. Hellerstein, William J. Evans, Alison D. Gernand, M. Munirul Islam, Steven A. Abrams, Jennifer Harrington, Edna Nyangau, Daniel E. Roth, Karen M. O’Callaghan

**Affiliations:** 1 Department of Nutritional Sciences, University of Toronto, Toronto, ON Canada; 2 Centre for Global Child Health, Hospital for Sick Children, Toronto, ON Canada; 3 Nutrition and Clinical Services Division, International Centre for Diarrhoeal Disease Research, Bangladesh (icddr,b), Dhaka, Bangladesh; 4 Faculty of Kinesiology and Physical Education, University of Toronto, Toronto, ON Canada; 5 Department of Nutritional Sciences and Toxicology, University of California, Berkeley, Berkeley, CA USA; 6 Department of Nutritional Sciences, The Pennsylvania State University, University Park, PA USA; 7 Department of Pediatrics, Dell Medical School at the University of Texas at Austin, Austin, TX USA; 8 Department of Pediatrics, Women’s and Children’s Health Network and University of Adelaide, Adelaide, SA Australia; 9 Division of Paediatric Medicine, Department of Paediatrics, Hospital for Sick Children, Toronto, ON Canada; 10 Department of Nutritional Sciences, King’s College London, London, UK

## Abstract

**Background:**

Given limited experience in applying the creatine-(methyl-D_3_) (D_3_Cr) dilution method to measure skeletal muscle mass (SMM) in young children, the feasibility of deployment in a fielding setting and performance of the method was assessed in a cohort of 4-year-old children in Dhaka, Bangladesh.

**Methods:**

Following D_3_Cr oral dose (10 mg) administration, single fasting urine samples were collected at 2–4 days (*n* = 100). Twenty-four-hour post-dose collections and serial spot urine samples on days 2, 3 and 4 were obtained in a subset of participants (*n* = 10). Urinary creatine, creatinine, D_3_Cr and D_3_-creatinine enrichment were analyzed by liquid chromatography–tandem mass spectrometry. Appendicular lean mass (ALM) was measured by dual-energy x-ray absorptiometry and grip strength was measured by a hand-held dynamometer.

**Results:**

SMM was measured successfully in 91% of participants, and there were no adverse events. Mean ± SD SMM was greater than ALM (4.5 ± 0.4 and 3.2 ± 0.6 kg, respectively). Precision of SMM was low (intraclass correlation = 0.20; 95% CI: 0.02, 0.75; *n* = 10). Grip strength was not associated with SMM in multivariable analysis (0.004 kg per 100 g of SMM; 95% CI: −0.031, 0.038; *n* = 91).

**Conclusions:**

The D_3_Cr dilution method was feasible in a community setting. However, high within-child variability in SMM estimates suggests the need for further optimization of this approach.

**Impact:**

- The D3-creatine (D3Cr) stable isotope dilution method was considered a feasible method for the estimation of skeletal muscle mass (SMM) in young children in a community setting and was well accepted among participants.
- SMM was weakly associated with both dual-energy x-ray absorptiometry-derived values of appendicular lean mass and grip strength.
- High within-child variability in estimated values of SMM suggests that further optimization of the D3Cr stable isotope dilution method is required prior to implementation in community research settings.

## Introduction

Skeletal muscle mass (SMM) is an important determinant of metabolic health across the lifecycle.^1–3^ Higher SMM has been associated with a lower risk of obesity, improved insulin sensitivity, and lower risks of disability, hip fracture, and mortality in later life.^4,5^ SMM is one of several components of lean mass (LM) (all non-bone, non-fat tissue, that includes muscle, connective and fibrotic tissue, and water);^6^ given its unique metabolic and contractile functions, differentiating SMM from LM is a key consideration in the examination of muscle-related health outcomes. Estimated muscle mass attainment in childhood influences peak muscle mass and cardiometabolic health in later life;^7,8^ however, longitudinal quantitative measures of true SMM in children are unavailable. There is a particular lack of evidence supporting methods to measure SMM in low-resource settings such as South Asia, where a high prevalence of linear growth faltering alongside rising obesity rates denotes the presence of the double burden of malnutrition.^9^ Given the increasing recognition of SMM as a marker of metabolic health, field-friendly methods for measurement of SMM in early life may be useful for testing interventions to enhance healthy growth and development in low-resource settings.

Various imaging modalities, including computerized axial tomography and magnetic resonance imaging (MRI), can yield precise estimates of SMM; however, these approaches are expensive and difficult to perform in large-scale studies, particularly in remote settings. Now widely used for body composition assessment, dual-energy x-ray absorptiometry (DXA) offers an advantage in terms of cost and feasibility;^10^ while this method involves minimal radiation exposure, the risk associated with single scanning is limited^11^ and DXA is considered safe in pediatric populations.^12^ DXA operates as a three-compartment model through the direct measure of bone and fat mass, with LM estimated by subtraction. Hence, it does not directly measure functional SMM.^6,10,13^ The non-invasive creatine-(methyl-D3) (D_3_Cr) dilution method determines total body creatine to estimate SMM; this method has been validated in adults using MRI,^14^ has been previously performed in infants^15^ and used in both large cohort studies^5,13,16,17^ and clinical trials.^18^ This method has also been shown to correlate with measures of muscle function in older adults, including grip strength.^13^ In a cohort of 4-year-old children in Dhaka, Bangladesh, this study examined the feasibility of using the D_3_Cr dilution method for the estimation of SMM in children in a community field setting, whereby the association of grip strength with D_3_Cr SMM was explored in comparison to LM estimates derived from DXA.

## Methods

### Design

“BONUSKids+” was a cross-sectional observational study nested within the BONe and mUScle health in Kids (BONUSKids; clinicaltrials.gov identifier #NCT03537443) study, which included follow-up of a subset of participants from the Maternal Vitamin D for Infant Growth (MDIG) trial (NCT01924013). A full description of the methods and primary outcomes for the MDIG trial^19,20^ and BONUSKids^21^ study has been previously reported. Briefly, MDIG was a double-blind, dose-ranging trial of maternal prenatal and postpartum vitamin D_3_ supplementation in Dhaka, Bangladesh (*n* = 1300), for which the primary outcome was infant linear growth at 12 months of age. The BONUSKids study involved examination of the prenatal and postpartum intervention on DXA-derived measures of bone mineral content, areal bone mineral density and body composition (lean and fat mass), as well as grip strength at 4 years of age (*n* = 642). All postnatal follow-up assessments for the MDIG trial were completed in March 2018 when infants reached 2 years of age; data collection for the BONUSKids study took place between October 2018 and February 2020.

Eligibility criteria for the BONUSKids study included availability for participation at 48 ± 3 months of age, ability to ambulate without assistance (i.e., not wheelchair bound or supported by an orthopedic cast), and absence of a diagnosed developmental disorder that would limit feasible DXA scanning. In addition, children who required texture modification of liquids and/or experienced difficulty swallowing due to a diagnosed neurological disorder were considered ineligible for participation in BONUSKids+ study activities. To ensure maximum absorption of the ingested isotope, enrollment in BONUSKids+ was postponed for otherwise eligible children if the child had experienced diarrhea or vomiting in the preceding 24 h; enrollment was re-attempted once the child had been symptom-free for 24 h.

Given limited prior studies describing the use of the D_3_Cr method among children in a community setting, we targeted a sample of 100 participants, enrolled in parallel to the BONUSKids study, such that data collection took place on the same day or within 1 month of primary BONUSKids study activities. All data collection for the BONUSKids+ study was completed between October and December 2019. Since the accuracy of the D_3_Cr dilution method is dependent upon complete absorption of the tracer dose (D_3_Cr) for transport to skeletal muscle, the first 10 participants were simultaneously enrolled in a 3-day pilot study to determine: (i) whether any D_3_Cr was excreted into urine following ingestion (defined herein as “spillage”), and; (ii) whether a steady-state ratio of D_3_-labeled creatinine to total creatinine (enrichment) was reached.

### Data collection

General health and household sociodemographic data were collected via interviewer-administered questionnaires at the 4-year visit. Asset index, as a proxy for socioeconomic status, was determined by claimed ownership of household items in the MDIG trial and generated using principal component analysis;^20^ the quintile assigned to each participant, therefore, reflects the asset index relative to the entire MDIG trial cohort and is not specific to participants of the present follow-up study. Full details of the methodology used to measure anthropometry, body composition and grip strength have been reported elsewhere.^21^ In brief, height was measured to the last completed 1 mm using a portable stadiometer (Leicester Height Measure, Chasmors, London, UK). Weight was measured to the nearest 50 g on a digital scale (Seca 874, Seca, Germany). Anthropometric *z*-scores (height, weight and BMI-for-age) were calculated in accordance with the WHO child growth standards.^22^ Grip strength was measured to the nearest 0.1 kg using a hand-held digital dynamometer (Jamar, Patterson Medical). All measurements were conducted in a seated position with the active arm resting at a 90-degree angle, in line with standardized approaches.^23^ Three readings were obtained from each hand, alternating between right and left hands, leaving a 30-s rest between measurements to prevent fatigue. Both the maximum and arithmetic mean of all attempted measurements was calculated; maximum grip strength reflected the single highest value of the left or right hand across all attempts, and was used for main analyses. Fat and LM was examined by DXA using a narrow-angle fan-beam DXA scanner (Lunar Prodigy Advance; GE Healthcare, Madison, WI) in the enhanced analysis mode on enCore software v16.0. Quality of the total-body-less-head (TBLH; sub-cranial skeleton from the base of neck to feet) DXA images were assessed independently for alignment and motion artifact, as previously described.^21^ The primary measure of appendicular lean mass (ALM) was calculated as the sum of LM of the four extremities (right and left arms; right and left legs) and expressed in kg. Average dietary energy and protein intakes were obtained from two non-consecutive interviewer-administered 24-h dietary recalls (Supplementary Methods).

### Oral administration of D_3_Cr

A single 10 mg (range: 9.2–9.9 mg) isotopic dose of D_3_Cr was provided to all participants, reflecting approximately 0.7 mg D_3_Cr per mean kg body weight (14.5 kg), and equating to a range of 0.4–1.0 mg/kg based on the maximum (25.6 kg) and minimum (10.4 kg) body weight, respectively, of the study cohort. The D_3_Cr dose was prepared, measured and packaged into individual vials by Cambridge Isotope Laboratories Inc. (Andover, MA) and transported in the anhydrous form to the International Centre for Diarrhoeal Disease Research, Bangladesh (icddr,b). Vials were stored at 2–8 °C until use.

The D_3_Cr dose solution was prepared for administration using 5–10 ml of water, a maximum of 2 h before ingestion. The dose was thoroughly dissolved in solution using an electric vortex. Participants were aided by study personnel to drink the whole solution directly from the vial. The empty vial was rinsed three times with 5–10 ml of water, which was consumed by each participant to ensure complete delivery of the dose.

### Urine collection

Continuous 24-h urine samples were collected for all 10 participants of the pilot study, who were admitted overnight to a dedicated clinical research unit at icddr,b. Urine collection began from the first void following oral D_3_Cr ingestion. Urine was collected into clean plastic potties and transferred to sterile urine collection containers for maintenance at 2–8 °C until frozen storage. The volume of pooled 24-h samples from each participant was weighed using an electronic balance (EK600i-600, AND) and 1 ml aliquots were stored at −20 °C until shipment to the University of California, Berkeley (UCB) for analysis.

Single fasting spot urine samples were collected by caregivers in the home upon first void after waking on days 2, 3 and 4 post-dose ingestion for participants of the pilot study (*n* = 10). One fasting urine sample was collected from all other participants (*n* = 90) within 2–4 days post-dose ingestion. Preference was given to collection on day 2 to permit additional sample collection on day 3 or 4, if required, since we expected achievement of steady-state enrichment of D_3_-creatinine (D_3_Crn) in urine within ~48 h.^14^ Second void fasting urine samples were collected if the first void after waking was missed. Samples were considered non-fasting if a creatine-containing food or beverage was consumed <8 h prior to urine collection. Caregivers were instructed to store urine samples in a cool area or refrigerator, if available, until same-day collection by study personnel, after which the sample was placed at 2–8 °C. Urine samples were stored immediately post processing at −20 °C until analysis.

### Laboratory analyses

Serum C-reactive protein (CRP) was measured in non-fasting venous blood samples by automated spectrophotometry (Beckman Coulter AU series, Beckman Coulter Inc.) within 1 day of blood collection at the Clinical Biochemistry Laboratory at icddr,b. Batched analysis of urinary total creatine, total creatinine, D_3_Cr and D_3_Crn was performed by liquid chromatography–tandem mass spectrometry (LC-MS/MS) at UCB using a standardized method that has been validated in adults,^14^ infants^24^ and children.^25^ The D_3_Crn enrichment ratio was defined as the molar ratio of D_3_Crn to total creatinine. One hundred microliter aliquots of urine were processed for D_3_Cr concentration and D_3_Crn enrichment using methods previously described.^26^ For D_3_Cr concentration, 100 μl of urine from the 24-h collection or standards for the standard curve was added to 100 μl internal standard and 200 μl acetonitrile. Samples were centrifuged to precipitate proteins, and aliquots of the supernatant were diluted 50-fold using 70% acetonitrile for LC-MS/MS analysis. Mass spectrometry was performed on a Sciex 6500 QTRAP (AB Sciex LLC, Framingham, MA) operating in multiple reaction monitoring mode. To determine the enrichment of D_3_Crn, 100 μl of urine from the fasting spot urine was processed as described above and quantitation was performed using a standard curve for D_3_Crn enrichment that spanned from 0 to 0.58% and measured multiple reaction monitoring transitions (114.1/44.1) corresponding to the M0 peak of Crn and 117.1/47.1 which corresponds to D_3_Crn. Samples were run in duplicate and average values were reported; CVs of duplicate analysis were <3%.

### Estimation of skeletal muscle mass

SMM was estimated from total body creatine pool size and calculated using a previously defined equation.^14^ Total muscle mass was determined based on the concentration of creatine in muscle mass (4.3 g/kg muscle),^27^ using the following formula:

$${{{{{{{\mathrm{Total}}}}}}}}\,{{{{{{{\mathrm{muscle}}}}}}}}\,{{{{{{{\mathrm{mass}}}}}}}}\,{{{{{{{\mathrm{estimate}}}}}}}}\,\left( {{{{{{{{\mathrm{kg}}}}}}}}} \right) = \frac{{{{{{{{{\mathrm{Creatine}}}}}}}}\,{{{{{{{\mathrm{pool}}}}}}}}\,{{{{{{{\mathrm{size}}}}}}}}\,\left( {{{{{{{\mathrm{g}}}}}}}} \right)}}{{4.3{{{{{{{\mathrm{g}}}}}}}}/{{{{{{{\mathrm{kg}}}}}}}}\,{{{{{{{\mathrm{muscle}}}}}}}}}}$$

Assuming 98% of creatine is stored in SMM:^14^

$${{{{{{{\mathrm{SMM}}}}}}}}\,\left( {{{{{{{{\mathrm{kg}}}}}}}}} \right) = {{{{{{{\mathrm{total}}}}}}}}\,{{{{{{{\mathrm{muscle}}}}}}}}\,{{{{{{{\mathrm{mass}}}}}}}}\left( {{{{{\mathrm{{kg}}}}}}} \right) \times 98\%$$

Participants were excluded from the SMM analyses if they had incomplete D_3_Cr dose ingestion, SMM values ≥50% of body weight, or a non-fasting urine sample (Supplementary Fig. 1).

### Statistical analyses

Distributions for continuous variables were visually inspected using histograms, kernel density plots and boxplots. Data were reported as means ± SD and frequencies (percentages), where appropriate. Participant characteristics were summarized collectively for the full cohort, and separately by sex. Given the nested study design of the BONUSKids+ study, sociodemographic characteristics are reported separately for the BONUSKids+ and primary BONUSKids cohorts to assess the external validity of the study findings. Bivariate relationships between variables were assessed using scatterplots and LOWESS curves. The strength of the pairwise relationships was assessed using Pearson or Spearman correlation coefficients, as appropriate.

To evaluate the feasibility of the D_3_Cr dilution method for use in field settings, the following metrics were calculated: proportion of complete doses ingested, percentage of adequate fasting urine samples, percentage of protocol deviations (i.e., adherence to fasting, collection of urine sample within designated timeframes), and presence of urinary “spillage” of the tracer dose.

To assess the reliability of the SMM measure, the intraclass correlation coefficient (ICC) was calculated from the repeated measurements of SMM in the 10 participants of the pilot study using a mixed effects model with a fixed effect of time between D_3_Cr dose and urine collection and a participant-level random intercept. In addition, within-participant CV was calculated by determining the SD/mean ratio separately for each participant and calculating the arithmetic mean of individual ratios across all pilot study participants.^28^ Between-participant CV was calculated by determining the group SD divided by the arithmetic group mean SMM.

Assessment of steady-state D_3_Cr enrichment was determined by visual inspection of the longitudinal measures of D_3_Cr using measures obtained from three spot urine collections over 3 days post-dose ingestion in the pilot study and by estimating the association of SMM with time since dose ingestion using linear regression for the combined data of SMM measures from all participants. As three spot urine samples were collected in the pilot study, data for the combined analysis was based on urine samples collected on day 2 post-dose ingestion.

Multivariable regression models were constructed to assess the association between maximum grip strength and each of the seven body composition measures: (i) SMM, (ii) ALM, (iii) upper extremities LM, (iv) lower extremities LM, (v) TBLH LM, (vi) BMI-for-age *z*-score, (vii) height, and (viii) weight. Models were adjusted for the following covariates which were considered a priori as possible confounders based on directed acyclic graphs: participant sex (male/female), age (months), height (cm), BMI-for-age *z*-score, maternal height (cm), maternal education, household asset index quintile, and the vitamin D supplementation group assigned to the participant’s mother at enrollment to the MDIG trial.^19^ To assess potential confounding by body size, adjustment for BMI-for-age *z*-score was used to avoid multicollinearity between height and weight.

In sensitivity analysis, regression models were fitted using average grip strength to provide evidence of the robustness of conclusions from the primary measure of maximum grip strength. We also limited the analysis to children with complete data for SMM, ALM and grip strength. Further analysis included stratification by sex to determine whether associations between grip strength and SMM, ALM and other anthropometric measures differed between boys and girls. In post hoc analysis, multivariable regression models exploring associations with grip strength were repeated with alternative expressions of SMM, ALM, and TBLH LM: SMM as a percentage of body weight, ALM as a percentage of body weight, TBLH LM divided by height squared, and ALM divided by height squared. Planned subgroup analysis involved the exclusion of participants with a CRP concentration >5 mg/l^29^ to account for inflammatory states that may impair absorption of D_3_Cr; however, this analysis was not performed as few participants presented with elevated CRP.

Statistical analyses were performed using STATA v17.0 (College Station, TX).

## Results

A total of 100 4-year-old children were enrolled with a nearly even distribution of boys and girls (Table 1). All 100 participants of the BONUSKids+ study attempted consumption of the D_3_Cr solution. No adverse event at dose administration was observed. Urine collections were completed for all participants at the targeted time points. SMM data from 9 children administered the D_3_Cr isotope were excluded from SMM analyses due to incomplete dosing (*n* = 5) (as defined by minimal loss of dose due to dribble during administration), a non-fasting urine sample (*n* = 2) and acknowledged overestimation of SMM when values exceeded 50% of body weight (*n* = 2), resulting in data from 91 participants included in primary analyses. Four participants were excluded from analyses involving ALM due to an incomplete or unusable DXA scan (Supplementary Fig. 1). There was evidence that participants of BONUSKids+ had lower enrollment from the highest socioeconomic status quintile compared to the larger BONUSKids study cohort (Supplementary Table 1). There was an uneven distribution of the assigned maternal vitamin D supplementation group at enrollment; however, other baseline characteristics were similar between the BONUSKids+ participants and the BONUSKids cohort (Supplementary Table 1).

**Table 1:** Characteristics of children in Dhaka, Bangladesh for whom skeletal muscle mass was estimated using the D_3_-creatine dilution method.

Participant characteristics	Total	Boys	Girls
	*n* = 100	*n* = 52	*n* = 48
Age (months)^a^	49.0 (0.4)	49.0 (0.4)	49.1 (0.4)
Height (cm)^a^	98.5 (4.3)	99.7 (4.8)	97.2 (3.3)
Weight (kg)^a^	14.5 (2.5)	15.2 (2.8)	13.8 (1.7)
Height-for-age *z*-score^a^	−1.2 (1.0)	−1.0 (1.1)	−1.4 (0.8)
BMI-for-age *z*-score^a^	−0.4 (1.1)	−0.2 (1.2)	−0.6 (0.9)
D_3_Cr SMM (kg)^a,b^	4.5 (0.9)	4.6 (1.0)	4.4 (0.8)
D_3_Cr SMM/total body weight (%)^a,b^	31.2 (4.9)	30.2 (4.8)	32.3 (4.8)
ALM (kg)^a,c^	3.2 (0.6)	3.5 (0.7)	3.0 (0.4)
ALM/total body weight (%)^a,c^	22.4 (1.9)	23.0 (1.8)	21.8 (1.8)
Appendicular lean mass/height^2^ (kg/m^2^)^a,c^	3.3 (0.4)	3.5 (0.5)	3.2 (0.3)
TBLH fat mass (kg)^a,c^	4.1 (1.4)	4.1 (1.7)	4.1 (1.0)
TBLH lean mass (kg)^a,c^	8.4 (1.3)	9.0 (1.4)	7.8 (0.9)
TBLH lean mass/height^2^ (kg/m^2^)^a,c^	8.6 (0.8)	9.0 (0.8)	8.3 (0.6)
Upper extremities lean mass (kg)^a,c^	0.7 (0.2)	0.8 (0.2)	0.6 (0.1)
Lower extremities lean mass (kg)^a,c^	2.5 (0.5)	2.7 (0.6)	2.3 (0.3)
Maximum hand-grip strength (kg)^a,d^	4.6 (1.3)	4.7 (1.3)	4.5 (1.3)
Average hand-grip strength (kg)^a,e^	3.6 (1.1)	3.8 (1.1)	3.5 (1.0)
Average protein intake (g/day)^a,f^	37.2 (14.6)	39.0 (15.3)	35.1 (13.8)
Average energy intake (kcal/day)^a,f^	1201 (375)	1260 (411)	1138 (324)
Average protein energy percentage^a,f,g^	12.4 (3.1)	12.5 (3.0)	12.2 (3.1)
C-reactive protein below LLOQ^h^, *n* (%)	91 (96)	47 (96)	44 (96)
Household asset index quintile ^i^,*n* (%)			
1 (lowest)	22 (22)	11 (21)	11 (23)
2	24 (24)	15 (29)	9 (19)
3	21 (21)	8 (15)	13 (27)
4	21 (21)	10 (19)	11 (23)
5 (highest)	12 (12)	8 (15)	4 (8)
Maternal height (cm)^a,j^	150.9 (5.4)	151.5 (4.9)	150.3 (5.8)
Maternal BMI category^k^, *n* (%)			
Normal or underweight (<25 kg/m^2^)	42 (43)	22 (43)	20 (42)
Overweight (25–<30 kg/m^2^)	37 (37)	20 (39)	17 (35)
Obese (≥30 kg/m^2^)	20 (20)	9 (18)	11 (23)
Maternal level of education^j^, *n* (%)			
Secondary incomplete or less	80 (80)	38 (73)	42 (88)
Secondary complete or higher	20 (20)	14 (27)	6 (12)
Maternal prenatal:postnatal vitamin D supplementation dose (IU/week), *n* (%)			
0:0	24 (24)	7 (13)	17 (35)
2400:0	21 (21)	13 (25)	8 (17)
16,800:0	25 (25)	14 (27)	11 (23)
28,000:0	16 (16)	6 (12)	10 (21)
28,000:28,000	14 (14)	12 (23)	2 (4)

*ALM* appendicular lean mass measured by dual energy X-ray absorptiometry, *D*_*3*_*Cr SMM* skeletal muscle mass measured by D_3_-creatine dilution method, *TBLH* total-body-less-head.

^a^Presented as mean (SD) (all such values).

^b^*n* = 91, 5 children consumed incomplete D_3_Cr dose, 2 children provided a non-fasting urine sample, 2 children had implausible SMM (SMM > 50% of body weight).

^c^*n* = 96; 2 children did not participate in the dual-energy x-ray absorptiometry scan, 2 scans were deemed unusable due to excess motion.

^d^Maximum hand-grip strength is the maximum value of all completed attempts.

^e^Average hand-grip strength is the arithmetic mean of all attempts from both hands.

^f^*n* = 99; dietary intake values for 1 child were considered implausible due to under-reporting (<500 kcal/d).

^g^Average protein energy percentage was calculated as the energy equivalent of the average daily protein intake in kcal (1 g protein = 4 kcal) divided by the average daily energy intake in kcal, multiplied by 100.

^h^LLOQ (lower limit of quantification) for C-reactive protein was 1.6 mg/L, *n* = 95.

^i^Determined at mother’s enrollment in the Maternal Vitamin D for Infant Growth (MDIG) trial by claimed ownership of specific household items and computed using principal component analysis.

^j^Maternal height and level of education collected at the MDIG trial enrollment visit were substituted for 2 participants whose mothers did not attend the BONUSKids+ study visit.

^k^*n* = 99; weight was not measured for 11 mothers, BMI from 12 months post-partum was substituted where available.

**Fig. 1:**
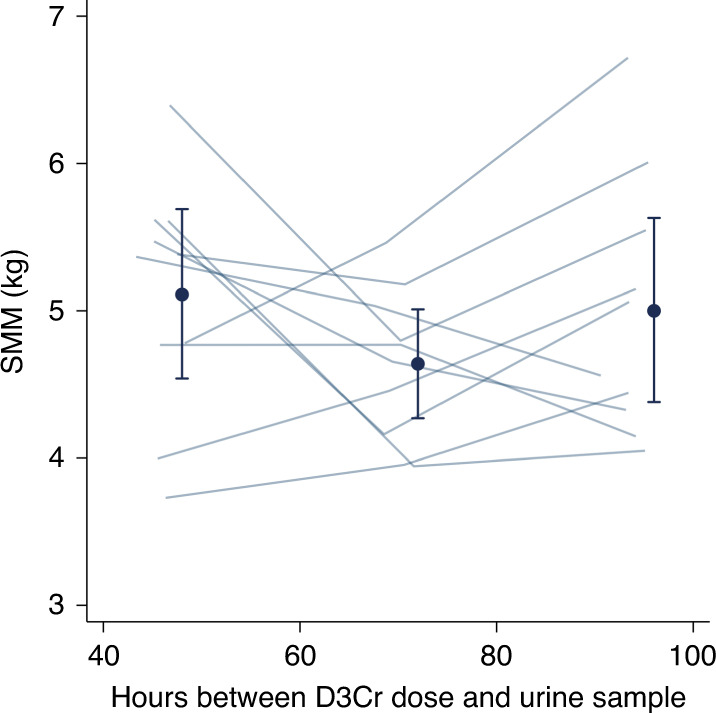
Skeletal muscle mass (SMM) estimates based on urine samples collected at different times elapsed since D_3_-creatine (D_3_Cr) dose ingestion in participants of the pilot study (*n* = 10). Each line represents an individual participant. Three measurements of SMM were conducted at 2, 3, and 4 days post-D_3_Cr dose ingestion. Filled circles represent mean SMM (kg) with 95% CIs indicated by the vertical bars when measurements were grouped into 2-, 3-, and 4-day urine collection categories (43–48, 68–72, and 91–95 h after dose, respectively).

D_3_Cr “spillage,” defined as detectable D_3_Cr in the post-dose 24-h urine collection, was not detected for any participants of the pilot study. Therefore, no correction factor was applied in the calculation of SMM. Visual inspection of the measured SMM plotted against time since dose ingestion suggested substantial dispersion of the data within each child when compared to the dispersion of the data across all participants (Fig. 1). In a univariate mixed effect model with participant-specific random intercepts, the low ICC (0.20; 95% CI: 0.02, 0.75) indicated a large proportion of total variance was attributable to within-child variability, which was corroborated by the finding that the between-child CV (15%) was not substantially greater than the average within-child CV (8%).

Consistent with the primary BONUSKids study findings,^21^ mean height- and BMI-for-age *z*-scores at 4 years of age were low in the BONUSKids+ cohort (Table 1). Overall mean SMM was 4.5 ± 0.9 kg. LM was significantly higher in boys versus girls (9.0 ± 1.4 versus 7.8 ± 0.9 kg, respectively, *p* < 0.001), whereas the difference in SMM was smaller and not statistically significant (4.6 ± 1.0 versus 4.4 ± 0.8 kg, respectively, in boys and girls, *p* = 0.5). The mean relative proportion of SMM/kg body weight was 31.2 ± 4.9%. Grip strength was greater in boys than in girls (Table 1). Average daily protein intake (37.2 ± 14.6 g/d) was high relative to body weight, corresponding to 12% of total energy intake, which falls within the recommended range for this age group (10–20%).^30^ Although 100% of participants met or exceeded protein recommendations, up to 55% did not meet energy requirements (Table 1).

**Fig. 2:**
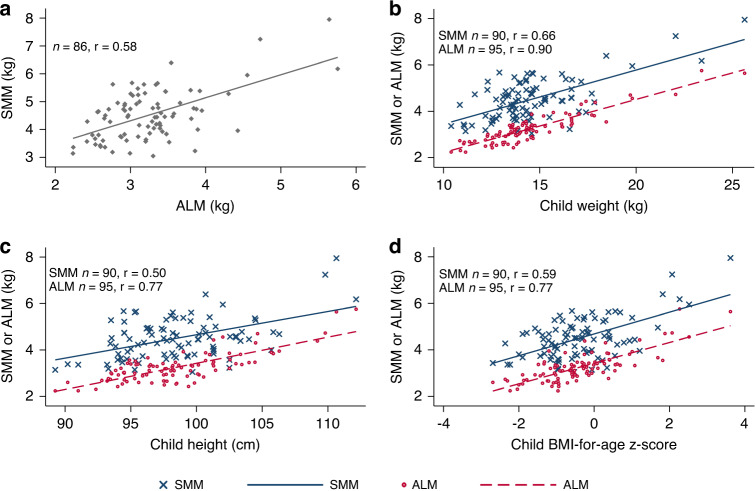
Linear associations between skeletal muscle mass (SMM) and selected anthropometric measurements. Associations of SMM measured using the D_3_-creatine (D_3_Cr) method with appendicular lean mass (ALM) measured using the dual energy X-ray absorptiometry (DXA) method (**a**), and associations of SMM or ALM with weight (**b**), height (**c**), and BMI-for-age *z*-score (**d**). Pearson correlation (*r*) is shown in each panel. Linear fit lines are shown as solid (SMM) or dashed (ALM) lines.

A linear relationship between SMM and ALM was observed (*r* = 0.57*, p* < 0.001). However, the correlation was fully attenuated when both variables were normalized to body weight (*r* = 0.05*, p* = 0.6). In nearly all participants, SMM accounted for a greater proportion of body weight than ALM; and, across the group, SMM tended to account for a lower proportion of weight at higher body weights, whereas the proportion contributed by ALM was unrelated to body weight (Supplementary Fig. 2). In comparison to SMM, ALM was more strongly correlated with selected anthropometric measurements (Fig. 2) and grip strength (Table 2 and Supplementary Fig. 3). In multivariable linear regression models adjusted for selected sociodemographic and body composition measures, the associations between grip strength and ALM, grip strength and TBLH LM, and between grip strength and SMM, were not statistically significant (Table 2). Inferences remained unchanged in sensitivity analyses when SMM, ALM, and TBLH LM were alternatively expressed by standardizing for other body size/composition measures (Table 2).

**Table 2:** Associations of maximum hand-grip strength with skeletal muscle mass, appendicular lean mass, and other body composition and anthropometric measures among 4-year-old children in Dhaka, Bangladesh.

Body composition or anthropometric variable	Pearson correlation			Unadjusted model^d^		Multivariable model^e^	
	*n*	*r*	p*	*β* (95% CI)	p*	*β* (95% CI)	p*
D_3_Cr SMM (100 g)^a^	91	0.33	0.001	0.046 (0.019, 0.074)	0.001	0.004 (−0.031, 0.038)	0.8
D_3_Cr SMM/total body weight (%)^a^	91	−0.11	0.3	−0.027 (−0.079, 0.026)	0.3	0.011 (−0.040, 0.061)	0.7
ALM (100 g)^a^	96	0.52	<0.001	0.106 (0.071, 0.142)	<0.001	0.074 (−0.016, 0.164)	0.1
ALM/height^2^ (kg/m^2^)^b^	96	0.44	<0.001	1.318 (0.774, 1.861)	<0.001	0.958 (−0.001, 1.916)	0.05
ALM/total body weight (%)^a^	96	0.21	0.04	0.146 (0.007, 0.284)	0.04	0.155 (0.021, 0.288)	0.02
Upper limb LM (100 g)^a^	96	0.54	<0.001	0.430 (0.291, 0.562)	<0.001	0.328 (0.052, 0.604)	0.02
Lower limb LM (100 g)^a^	96	0.50	<0.001	0.133 (0.086, 0.180)	<0.001	0.066 (−0.050, 0.182)	0.3
TBLH LM (100 g)^a^	96	0.51	<0.001	0.050 (0.032, 0.067)	<0.001	0.041 (−0.016, 0.098)	0.2
TBLH LM/height^2^ (kg/m^2^)^b^	96	0.41	<0.001	0.666 (0.359, 0.972)	<0.001	0.474 (−0.124, 1.073)	0.1
BMI-for-age *z*-score^c^	100	0.37	<0.001	0.449 (0.226, 0.671)	<0.001	0.268 (−0.009, 0.546)	0.06
Height (cm)^b^	100	0.45	<0.001	0.138 (0.084, 0.192)	<0.001	0.117 (0.045, 0.189)	0.002
Weight (kg)^c^	100	0.46	<0.001	0.245 (0.151, 0.340)	<0.001	0.158 (−0.042, 0.358)	0.1

*ALM* appendicular lean mass measured by dual X-ray absorptiometry, *LM* lean mass measured by dual energy X-ray absorptiometry, *D*_*3*_*Cr SMM* skeletal muscle mass measured by D_3_-creatine dilution method, *TBLH* total-body-less-head.

^a^Multivariable model includes child sex, age (months), height (cm), BMI-for-age *z*-score, maternal height (cm), maternal education, asset index quintile, and maternal MDIG vitamin D supplementation group.

^b^Multivariable model includes child sex, age (months), BMI-for-age z-score, maternal height (cm), maternal education, asset index quintile, and maternal MDIG vitamin D supplementation group.

^c^Multivariable model includes child sex, age (months), height (cm), maternal height (cm), maternal education, asset index quintile, and maternal MDIG vitamin D supplementation group.

^d^Estimates are interpreted as the average change in maximum hand-grip strength in kg for every 1-unit change in the body composition or anthropometric variable.

^e^Estimates are interpreted as the average change in maximum hand-grip strength in kg for every 1-unit change in the body composition or anthropometric variable, holding all other covariates constant.

*p < 0.05 is considered statistically significant.

Similar findings of positive Spearman correlations were found between dietary protein intake and both SMM (*rho* = 0.23, *p* = 0.03, *n* = 90) and ALM (*rho* = 0.19, *p* = 0.08, *n* = 88), although the observed relationships appeared non-linear (Supplementary Fig. 4). Acknowledging the plausible overestimation of dietary intakes and low between-day correlation of the protein intake estimates in the full sample (ICC = 0.23; 95% CI: 0.09, 0.46; *n* = 100), the association of dietary protein intake was not explored as an independent variable in the multivariable regression models to examine its association with SMM or ALM.

Both absolute height (cm) and upper limb LM showed moderate correlations with maximum grip strength (Table 2) and were positively associated with grip strength after adjustment for sociodemographic and anthropometric measures (Table 2). Inferences were unchanged in analyses using average grip strength (Supplementary Table 2) and when analyses were restricted to participants with complete data for SMM, ALM and grip strength (Supplementary Table 3). Comparing boys and girls, there was a similar association of grip strength with body composition measures in unadjusted models, but the direction and/or magnitude of the effect estimates differed between the sexes in multivariable models; in girls, the positive association of height with grip strength remained significant and an independent association was observed for both appendicular and lower limb LM, whereas in boys, only the positive association of upper limb LM with grip strength remained significant (Supplementary Table 4).

## Discussion

In this cohort of 4-year-old children in an urban community in Bangladesh, we demonstrated the application of the D_3_Cr dilution method as a field-friendly tool to quantify SMM based on the enrichment of D_3_Crn in urine. Contrary to our hypothesis, we did not find a statistically significant association between grip strength and D_3_Cr-derived SMM but rather report a stronger association of grip strength with DXA-derived ALM. However, this study exhibited high participant acceptability and compliance to the D_3_Cr method protocol, thereby supporting the feasibility of this method for future use in similar settings.

As described by Clark et al., the D_3_Cr dilution method leverages the unique pharmacokinetics of creatine,^31^ whereby an orally administered dose of D_3_Cr is rapidly absorbed and distributed to all skeletal muscle for conversion to D_3_Crn. SMM is subsequently calculated from the measurement of urinary D_3_Crn enrichment and total creatine pool size. The method relies on the complete uptake of D_3_Cr into skeletal muscle. Previous work^14^ in adults reported the presence of spillage of D_3_Cr in urine that necessitated the application of a correction factor to account for direct loss of the isotope within the first 24 h post ingestion.^14^ Consistent with data in premature infants,^15^ we did not find any evidence of D_3_Cr spillage in this study, confirming that a correction factor is unlikely to be required when using the D_3_Cr method to quantify SMM in pediatric populations. Findings from the present analysis corroborate earlier work by Evans et al.,^15^ showing steady-state isotopic enrichment of D_3_Crn is apparent from 2 to 4 days post D_3_Cr dose ingestion. However, we observed high intra-child variability in SMM estimates based on serial urine samples collected from the subset of 10 participants of the pilot study. Although urine samples used in the analysis were collected in the fasting state, higher amounts of residual dietary creatinine in some samples may have diluted the D_3_Crn enrichment in urine and, hence, led to an overestimation of SMM. Strict control of the pre-fasting meal, such as restriction to low-creatinine foods, as well as a collection of a second void fasting urine sample, may overcome this issue in future applications. However, this approach would be less convenient in community settings and among pediatric participants for whom extended periods of fasting may be unfeasible.

DXA-derived ALM has been shown to explain >98% of the variance in gold-standard MRI-derived SMM in children.^32^ In line with prior research in adults,^14^ the present study confirms that TBLH LM derived from DXA overestimates muscle mass compared to total body D_3_Cr-derived SMM. Conversely, ALM values were lower than total body SMM but still reflected the majority of total body SMM, which is consistent with the understanding that the extremities constitute a substantial portion of total body SMM.^10,33^ However, mean D_3_Cr-derived SMM values in our study were higher than would be expected (~2.43 kg) based on predictive equations developed in the United States (where SMM = (1.115 × ALM) − 1.133).^32^ Although these equations may not be generalizable across diverse populations, particularly since height is an important correlate of muscle mass,^34^ the D_3_Cr-derived SMM values reported here likely over-estimate true SMM in this population in which there is a high prevalence of linear growth faltering.^35^ The creatine content of skeletal muscle is known to vary by fiber type, with greater creatine levels in fast twitch compared to slow twitch fibers.^36,37^ Although the use of a constant value (4.3 g/kg) is considered applicable to adults of varying ages,^14^ this conversion factor may not be appropriate for children who have a comparably lower proportion and cross-sectional area of type II muscle fibers.^38^

We are not aware of any prior studies that have directly compared D_3_Cr-derived SMM and DXA-derived ALM in young children. The correlation between D_3_Cr-derived SMM with DXA-derived ALM was lower in the present study (*r* = 0.58) than previously reported in older men (*r* = 0.68)^13^ and men of mixed ages (*r* = 0.957).^14^ Furthermore, in contrast to findings in older adults,^39^ we observed a stronger correlation between grip strength and ALM compared to the relationship of grip strength with SMM, even though SMM was expected to more closely represent the contractile muscle compartment. We acknowledge the imprecision of the SMM values, as evidenced by the substantial within-child variation in SMM in the pilot study, which likely attenuated the strength of the correlation between SMM and grip strength.

Although grip strength is considered a valid proxy for overall muscle strength,^40^ the effect estimates for the association of upper limb LM with maximum grip strength was of the greatest magnitude relative to other segments of LM explored. This finding was expected considering the force generated in hand-grip strength is likely to be primarily a function of upper-body muscle mass.^34^ As SMM represents a total body measurement and cannot be partitioned into compartmental segments of appendicular LM, it is possible that the absence of a relationship between SMM and grip strength is explained by the inability to differentiate functional muscle mass of the upper and lower extremities rather than a consequence of imprecise SMM values. Our findings, therefore, suggest DXA-derived measures of LM are a useful indicator of muscle function that perform well compared to total-body D_3_Cr-derived SMM. While the D_3_Cr dilution method offers the advantage of portable assessment conducted within the participant’s home or field site, the cost and technical expertise required to perform LC-MS/MS is an additional consideration that may preclude its implementation in some settings. However, relative material and implementation costs will vary by study setting, and a cost-analysis comparison was not performed in the present study.

This study presents novel findings for SMM data among young children in urban Bangladesh using the D_3_Cr dilution method. The collection of continuous 24-h urine samples in a subset of participants enabled examination of D_3_Cr “spillage” following D_3_Cr dose ingestion, of which the findings may directly inform the application of this method in future studies. However, several limitations should be acknowledged. Only one fasting urine sample was available for all participants, and hence, we could not confirm the timing at which a plateau in D_3_Crn enrichment was reached. Since environmental enteric dysfunction (subacute inflammatory condition of the small intestinal mucosa with unclear etiology) is prevalent in Bangladesh,^41–44^ chronic low-grade inflammation and compromised intestinal barrier may have impaired creatine absorption. As the eligibility criteria ensured the absence of symptoms reflecting acute or chronic diarrhea and most participants presented with low circulating CRP concentrations, we do not believe underlying intestinal disorders to have biased our results. We did not collect data on physical activity, and acknowledge the imprecision of the nutrient data (energy and protein); therefore, neither activity level nor dietary intake was accounted for in multivariable models. Greater precision of the effect estimates may have been observed if such variables were included. We did not quantify nor standardize the hydration status of participants in this study. However, the impact of hydration on variability in DXA-derived LM is minor and predominantly influences measures of LM that include the trunk.^45^ The high precision of DXA for the estimation of LM has been previously established.^46^ To limit radiation exposure, multiple DXA scans were therefore not performed, and hence, intra-individual comparison of LM measures was not conducted. Our assessment of feasibility was limited in scope and further consideration of implementation costs is required. Lastly, we acknowledge the relatively selective nature of this cohort in addition to the small sample size, which limits the generalizability of our findings with respect to the broader population of children in Dhaka or elsewhere.

## Conclusion

This study demonstrated the feasibility of the D_3_Cr dilution method to measure SMM in young children in Dhaka, Bangladesh, thereby providing an empirical basis for the future application of this method in similar research settings. However, given the high within-child variability and weak association of SMM with grip strength, the advantages of the D_3_Cr dilution method over proxy measures of SMM in this age group and setting remain uncertain.

## Supplementary information

Supplementary Material

## Data Availability

Data described in the manuscript, code book, and analytic code will be made available upon request to the authors. De-identified individual participant data will be provided for use in secondary data analyses approved by an independent research ethics board, and data requestors will be required to sign a data access agreement.

## References

[1] Orsso CE (2019). Metabolic implications of low muscle mass in the pediatric population: a critical review. Metabolism.

[2] Prado CM (2018). Implications of low muscle mass across the continuum of care: a narrative review. Ann. Med..

[3] Frontera WR, Ochala J (2015). Skeletal muscle: a brief review of structure and function. Calcif. Tissue Int..

[4] Cawthon PM (2009). Loss of hip BMD in older men: the osteoporotic fractures in men (MrOS) study. J. Bone Min. Res..

[5] Cawthon PM (2021). Muscle mass assessed by the D3-creatine dilution method and incident self-reported disability and mortality in a prospective observational study of community-dwelling older men. J. Gerontol. A Biol. Sci. Med. Sci..

[6] Heymsfield SB, Gonzalez MC, Lu J, Jia G, Zheng J (2015). Skeletal muscle mass and quality: evolution of modern measurement concepts in the context of sarcopenia. Proc. Nutr. Soc..

[7] McCarthy HD, Samani-Radia D, Jebb SA, Prentice AM (2014). Skeletal muscle mass reference curves for children and adolescents. Pediatr. Obes..

[8] Brown LD (2014). Endocrine regulation of fetal skeletal muscle growth: impact on future metabolic health. J. Endocrinol..

[9] Popkin BM, Corvalan C, Grummer-Strawn LM (2020). Dynamics of the double burden of malnutrition and the changing nutrition reality. Lancet.

[10] Heymsfield SB, Adamek M, Gonzalez MC, Jia G, Thomas DM (2014). Assessing skeletal muscle mass: historical overview and state of the art. J. Cachexia Sarcopenia Muscle.

[11] Law M (2019). Cumulative effective dose and cancer risk of pediatric population in repetitive whole-body scan using dual-energy X-ray absorptiometry. J. Clin. Densitom..

[12] Gordon CM (2008). Dual energy X-ray absorptiometry interpretation and reporting in children and adolescents: the 2007 ISCD Pediatric Official Positions. J. Clin. Densitom..

[13] Cawthon PM (2019). Strong relation between muscle mass determined by D3-creatine dilution, physical performance, and incidence of falls and mobility limitations in a prospective cohort of older men. J. Gerontol. A Biol. Sci. Med. Sci..

[14] Clark RV (2014). Total body skeletal muscle mass: estimation by creatine (methyl-D3) dilution in humans. J. Appl. Physiol. (1985).

[15] Evans, W. J. et al. D3-creatine dilution for the noninvasive measurement of skeletal muscle mass in premature infants. *Pediatr. Res.* **89**, 1508–1514 (2021). 10.1038/s41390-020-01122-w 32919390

[16] Duchowny KA (2020). Association of change in muscle mass assessed by D3-creatine dilution with changes in grip strength and walking speed. J. Cachexia Sarcopenia Muscle.

[17] Cawthon PM (2009). Do muscle mass, muscle density, strength, and physical function similarly influence risk of hospitalization in older adults?. J. Am. Geriatr. Soc..

[18] Howard, E. E. et al. Effects of testosterone on mixed-muscle protein synthesis and proteome dynamics during energy deficit. *J. Clin. Endocrinol. Metab.* **107**, e3254–e3263 (2022). 10.1210/clinem/dgac295 35532889

[19] Roth DE (2015). Maternal vitamin D supplementation during pregnancy and lactation to promote infant growth in Dhaka, Bangladesh (MDIG trial): study protocol for a randomized controlled trial. Trials.

[20] Roth DE (2018). Vitamin D supplementation in pregnancy and lactation and infant growth. N. Engl. J. Med..

[21] O’Callaghan KM (2022). Effect of maternal prenatal and postpartum vitamin D supplementation on offspring bone mass and muscle strength in early childhood: follow-up of a randomized controlled trial. Am. J. Clin. Nutr..

[22] World Health Organization. *WHO Child Growth Standards*. http://www.who.int/childgrowth/en/ (2006).

[23] Roberts HC (2011). A review of the measurement of grip strength in clinical and epidemiological studies: towards a standardised approach. Age Ageing.

[24] Scottoline, B. et al. Deuterated, D3-Creatine and heavy water dilution to measure total body muscle and lean mass in neonates: validation of a novel, non-invasive method. in *Pediatric Academic Societies Meeting* (2019).

[25] Evans, W. J. et al. Profoundly lower muscle mass and rate of contractile protein synthesis in boys with Duchenne muscular dystrophy. *J. Physiol.* **107**, e3254–e3263 (2022). 10.1113/JP282227 34569076

[26] Shankaran M (2018). Dilution of oral D3-Creatine to measure creatine pool size and estimate skeletal muscle mass: development of a correction algorithm. J. Cachexia Sarcopenia Muscle.

[27] Kreisberg RA, Bowdoin B, Meador CK (1970). Measurement of muscle mass in humans by isotopic dilution of creatine-14C. J. Appl. Physiol..

[28] Nybacka S (2019). Within- and between-subject variation in dietary intake of fermentable oligo-, di-, monosaccharides, and polyols among patients with irritable bowel syndrome. Curr. Dev. Nutr..

[29] World Health Organization. (2014). C-reactive Protein Concentrations as a Marker of Inflammation or Infection for Interpreting Biomarkers of Micronutrient Status.

[30] Institute of Medicine. (2005). Dietary Reference Intakes: Energy, Carbohydrate, Fiber, Fat, Fatty Acids, Cholesterol, Protein and Amino Acids.

[31] Wyss M, Kaddurah-Daouk R (2000). Creatine and creatinine metabolism. Physiol. Rev..

[32] Kim J (2006). Total-body skeletal muscle mass: estimation by dual-energy X-ray absorptiometry in children and adolescents. Am. J. Clin. Nutr..

[33] Snyder, W. S. et al. Report of the task group on reference man. *ICRP* (1984). 10.1016/0146-6453(79)90123-4 20863799

[34] Fricke O, Schoenau E (2005). Examining the developing skeletal muscle: why, what and how?. J. Musculoskelet. Neuronal. Interact..

[35] Bangladesh Demographic and Health Survey. *Ministry of Health and Family Welfare* (2017).

[36] Tesch PA, Thorsson A, Fujitsuka N (1989). Creatine phosphate in fiber types of skeletal muscle before and after exhaustive exercise. J. Appl. Physiol. (1985).

[37] Sahlin K, Soderlund K, Tonkonogi M, Hirakoba K (1997). Phosphocreatine content in single fibers of human muscle after sustained submaximal exercise. Am. J. Physiol..

[38] Esbjornsson ME, Dahlstrom MS, Gierup JW, Jansson EC (2021). Muscle fiber size in healthy children and adults in relation to sex and fiber types. Muscle Nerve.

[39] Cegielski J (2022). The Combined Oral Stable Isotope Assessment of Muscle (COSIAM) reveals D-3 creatine derived muscle mass as a standout cross-sectional biomarker of muscle physiology vitality in older age. Geroscience.

[40] Wind AE, Takken T, Helders PJ, Engelbert RH (2010). Is grip strength a predictor for total muscle strength in healthy children, adolescents, and young adults?. Eur. J. Pediatr..

[41] Mahfuz M (2017). Bangladesh Environmental Enteric Dysfunction (BEED) study: protocol for a community-based intervention study to validate non-invasive biomarkers of environmental enteric dysfunction. BMJ Open.

[42] Richard, S. A. et al. Enteric dysfunction and other factors associated with attained size at 5 years: MAL-ED birth cohort study findings. *Am. J. Clin. Nutr.* **110**, 131–138 (2019). 10.1093/ajcn/nqz004 PMC6599740 31127812

[43] Billah SM (2019). Bangladesh: a success case in combating childhood diarrhoea. J. Glob. Health.

[44] Goto R, Mascie-Taylor CG, Lunn PG (2009). Impact of intestinal permeability, inflammation status and parasitic infections on infant growth faltering in rural Bangladesh. Br. J. Nutr..

[45] Horber FF, Thomi F, Casez JP, Fonteille J, Jaeger P (1992). Impact of hydration status on body composition as measured by dual energy X-ray absorptiometry in normal volunteers and patients on haemodialysis. Br. J. Radio..

[46] Mazess RB, Barden HS, Bisek JP, Hanson J (1990). Dual-energy x-ray absorptiometry for total-body and regional bone-mineral and soft-tissue composition. Am. J. Clin. Nutr..

