# Supplementary material for: Estimation of skeletal muscle mass in 4-year-old children using the D3-creatine dilution method
Source: Pediatr Res. 2023 Apr 10;94(3):1195–202. doi: 10.1038/s41390-023-02587-1 (PMC10444613; doi:10.1038/s41390-023-02587-1)
Supplement: Supplementary file 1 — Supplementary Material [file 41390_2023_2587_MOESM1_ESM.pdf]

Supplementary Material

Estimation of skeletal muscle mass in 4-year-old children using the D3-creatine dilution method

Aysha Sidiqi, Farzana Fariha, Shaila S Shanta, Alison Dasiewicz, Abdullah Al Mahmud, Daniel R Moore, Mahalakshmi Shankaran, Marc K Hellerstein, William J Evans, Alison D Gernand, M Munirul Islam, Steven A Abrams, Jennifer Harrington, Edna Nyangau, Daniel E. Roth and Karen M. O'Callaghan

Corresponding author:
Dr. Karen M. O'Callaghan
Department of Nutritional Sciences
King's College London
150 Stamford St
London SE1 9NH
United Kingdom
Email: karen.ocallaghan@kcl.ac.uk

Supplementary Methods. 1

List of Supplementary Tables

Supplementary Table 1. Characteristics of 4-year-old children in Dhaka, Bangladesh included in the BONUSKids study estimating body composition using the dual X-ray absorptiometry method and/or included in the BONUSKids+ sub-study using the D_3-creatine dilution method. 3

Supplementary Table 2. Associations of average hand-grip strength with skeletal muscle mass, appendicular lean mass, and other body composition and anthropometric measures among 4-year-old children in Dhaka, Bangladesh. 8

Supplementary Table 3. Associations of maximum hand-grip strength with skeletal muscle mass, appendicular lean mass, and other body composition and anthropometric measures among participants with complete data for skeletal muscle mass, appendicular lean mass and hand-grip strength. 9

Supplementary Table 4. Associations of maximum hand-grip strength with skeletal muscle mass, appendicular lean mass, and other body composition and anthropometric measures, stratified by sex, among 4-year-old children in Dhaka, Bangladesh. 10

List of Supplementary Figures

Supplementary Figure 1. Sample size of regression analyses. 2
Supplementary Figure 2. Body composition for the BONUSKids+ subsample with complete skeletal muscle mass (SMM) and appendicular lean mass (ALM) data. 5
Supplementary Figure 3. Associations of maximum hand-grip strength with skeletal muscle mass (SMM) and appendicular lean mass (ALM). 6
Supplementary Figure 4. Associations of average daily protein intake with skeletal muscle mass (SMM) and appendicular lean mass (ALM). 7

References 11

Supplementary Methods

Assessment of dietary intake

Dietary energy and protein intake was obtained from 2 non-consecutive interviewer-administered 24-hour dietary recalls. Estimated quantities of foods and beverages were reported by the participant's caregiver with the help of visual aids and models, and full recipes of meals made within the home were recorded as per the caregiver's recollection. Intakes of energy and protein were estimated using ESHA nutritional analysis software (Food Processor Nutrition Analysis Software, version 11.7, ESHA Research Inc., OR), to which the Food Composition Table (FCT) for Bangladesh[1] was integrated and used as the primary nutrient database. All data was entered independently by 2 study personnel, and discrepancies reconciled by a third investigator. Modifications to the nutrient database were conducted to include local recipes and the nutrient content from manufacturer food labels. Missing nutrient data were imputed using the UK food database or the United States Department of Agriculture (USDA) database, both of which were preloaded in the ESHA Food Processor software, as the required data of interest was not available from FCTs of neighbouring countries.

Average dietary energy and protein intakes were calculated as the mean value from each recall and expressed as kcal and grams per day, respectively. To assess within- versus between-child variability, we used a mixed effects linear model with random intercepts at the participant level and a fixed-effect to distinguish intakes on day 1 or 2, and estimated the model-derived intraclass correlation coefficient (ICC). An average daily energy intake of <500kcal/d or >3,500kcal/d was considered implausible[2], and hence participant data meeting such thresholds were excluded from analysis.

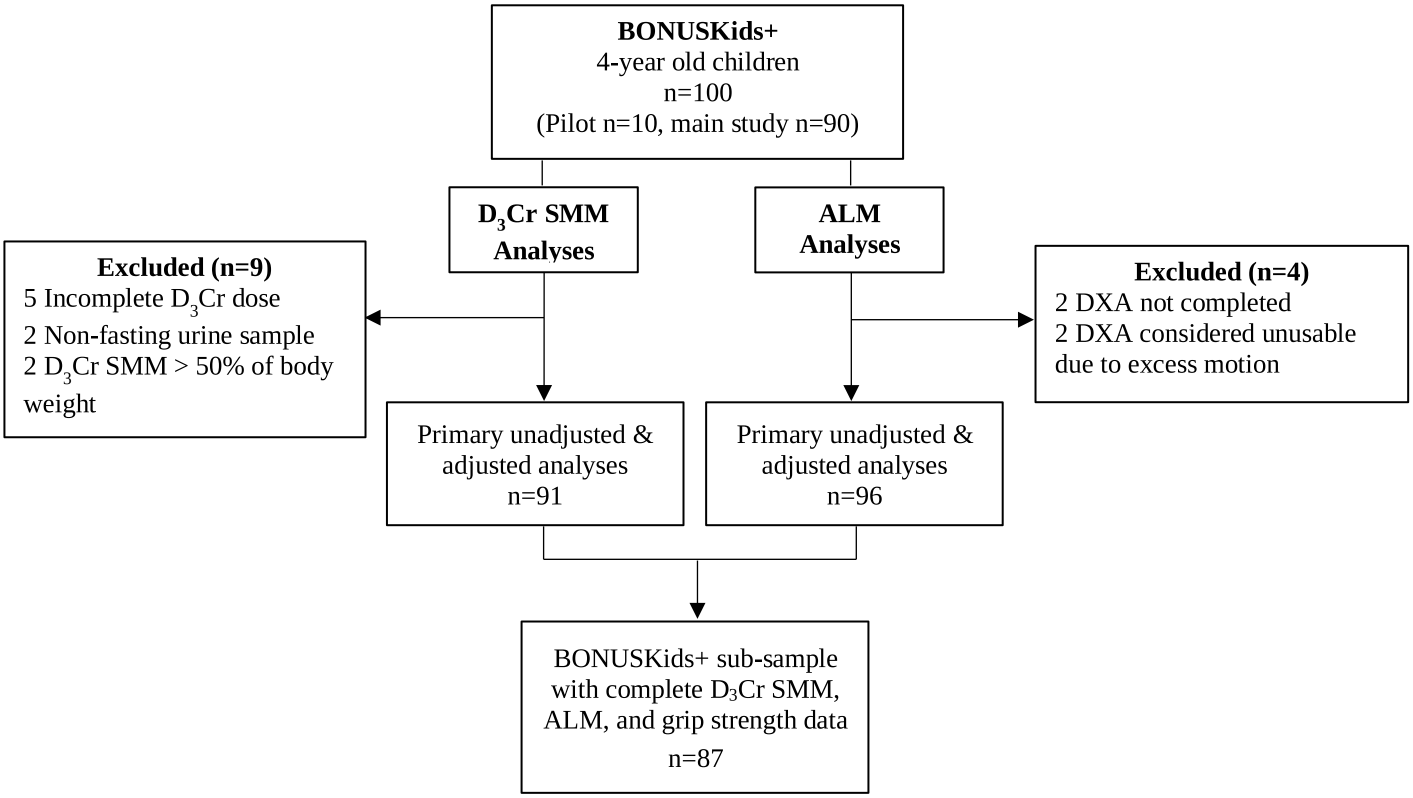

Supplementary Figure 1. Sample size of regression analyses.
ALM appendicular lean mass, *D_3Cr SMM* skeletal muscle mass measured by D_3-creatine dilution method, *DXA* dual-energy x-ray absorptiometry.

Supplementary Table 1. Characteristics of 4-year-old children in Dhaka, Bangladesh included in the BONUSKids study estimating body composition using the dual X-ray absorptiometry method and/or included in the BONUSKids+ sub-study using the D_3-creatine dilution method.

**Participant Characteristics**	**BONUSKids**	**BONUSKids+ (Included in D_3-Cr dilution method sub-study)**	**BONUSKids+ sub-sample with complete D_3Cr SMM, ALM, and grip strength data**
N	642	100	87
Age (months)[a]	49.6 (1.0)	49.0 (0.4)	49.0 (0.4)
Sex (male), *n (%)*	321 (50)	52 (52)	44 (51)
Height (cm)[a]	98.7 (4.4)	98.5 (4.3)	98.5 (4.3)
Weight (kg) [a]	14.4 (2.3)[b]	14.5 (2.5)	14.5 (2.5)
Height-for-age z-score[a,c]	-1.2 (1.0)	-1.2 (1.0)	-1.2 (1.0)
BMI-for-age z-score[a,c]	-0.5 (1.1)	-0.4 (1.1)	-0.4 (1.1)
D_3Cr SMM (kg)[a]	-	4.5 (0.9)	4.5 (0.9)
D_3Cr SMM / total body weight (%)[a]	-	31.2 (4.9)	31.3 (5.0)
ALM (kg)[a,d]	3.2 (0.5)	3.2 (0.6)	3.2 (0.6)
ALM / height^2 (kg/m^2)[a,d]	3.3 (0.4)	3.3 (0.4)	3.3 (0.4)
ALM / total body weight (%)[a,d]	22.4 (2.0)	22.4 (1.9)	22.3 (1.9)
TBLH fat mass (kg)[a,d]	3.9 (1.3)	4.1 (1.4)	4.1 (1.4)
TBLH lean mass (kg)[a,d]	8.4 (1.2)	8.4 (1.3)	8.4 (1.3)
TBLH lean mass / height^2 (kg/m^2)[a,d]	8.6 (0.7)	8.6 (0.8)	8.6 (0.8)
Upper extremities lean mass (kg)[a,d]	0.7 (0.1)	0.7 (0.2)	0.7 (0.2)
Lower extremities lean mass (kg)[a,d]	2.5 (0.4)	2.5 (0.5)	2.5 (0.5)
Maximum hand-grip strength (kg)[a,e]	4.5 (1.3)	4.6 (1.3)	4.6 (1.2)
Average hand-grip strength (kg)[a,f]	3.7 (1.2)	3.6 (1.1)	3.7 (1.0)
C-reactive protein below LLoQ[g], *n(%)*	499 (87)	91 (96)	79 (96)
Asset index quintile[h], *n (%)*			
1 (lowest)	117/640 (18)	22/100 (22)	20/87 (23)
2	126/640 (20)	24/100 (24)	22/87 (25)
3	130/640 (20)	21/100 (21)	19/87 (22)
4	139/640 (22)	21/100 (21)	16/87 (18)
5 (highest)	128/640 (20)	12/100 (12)	10/87 (12)
Maternal height (cm)[a,i]	151.0 (5.5)	150.9 (5.4)	151.2 (5.4)
Maternal BMI category[j], *n (%)*			
Normal or underweight (<25 kg/m^2)	264/615 (43)	42 (43)	37 (42)
Overweight (≥25 to <30 kg/m^2)	253/615 (41)	37 (37)	33 (38)
Obese (≥30 kg/m^2)	98/615 (16)	20 (20)	17 (20)
Maternal level of education[k], *n (%)*			
Secondary incomplete or less	501/642 (78)	80/100 (80)	71/87 (82)
Secondary complete or higher	141/642 (22)	20/100 (20)	16/87 (18)
Maternal prenatal:postnatal vitamin D supplementation dose (IU/week), *n (%)*			
0:0	121/642 (19)	24/100 (24)	19/87 (22)
2400:0	137/642 (21)	21/100 (21)	20/87 (23)
16800:0	130/642 (20)	25/100 (25)	23/87 (26)
28000:0	129/642 (20)	16/100 (16)	14/87 (16)
28000:28000	125/642 (20)	14/100 (14)	11/87 (13)

ALM appendicular lean mass measured by dual X-ray absorptiometry, *SMM* skeletal muscle mass measured by D_3-creatine dilution method, *TBLH* total body less head.
[a] Presented as mean (SD) (all such values).
[b] Weight not measured for 2 children, n = 640.
[c] Z-scores were standardised using the World Health Organization's age- and sex-specific growth curves.
[d] n = 599 participants had a usable DXA scan (no motion artifact or minor motion artifact). Of participants included in the D_3-creatine sub-study, n = 96 had a usable DXA scan.
[e] Maximum hand-grip strength is the maximum value of all completed attempts. In the primary BONUSKids study, n = 630.
[f] Average hand-grip strength is the arithmetic mean of all attempts from both hands. In the DXA study, n = 630.
[g] LLoQ (lower limit of quantification) for C-reactive protein was 1.6 mg/L; BONUSKids n = 572, BONUSKids+ n = 95, BONUSKids+ subsample n = 82.
[h] Determined at mother's enrolment in the Maternal Vitamin D for Infant Growth (MDIG) trial by claimed ownership of specific household items and computed using principal component analysis. The quintile assigned to each participant reflects the asset index relative to other MDIG participants and is not specific to participants of this follow-up study.
[i] Maternal height at baseline MDIG trial visit was substituted for 28 DXA study participants whose maternal height was not available at the BONUSKids study visit, 2 of which were also part of the D_3-creatine sub-study.
[j] BMI at 12-months postpartum was substituted for 36 mothers whose BMI was not available at the BONUSKids study visit; of these 36, 10 substitutions were for BONUSKids+ and 6 for the BONUSKids+ sub-sample.
[k] Maternal education at MDIG baseline visit was substituted for 17 participants whose maternal education was missing at the BONUSKids study visit, 2 of which were also part of BONUSKids+.

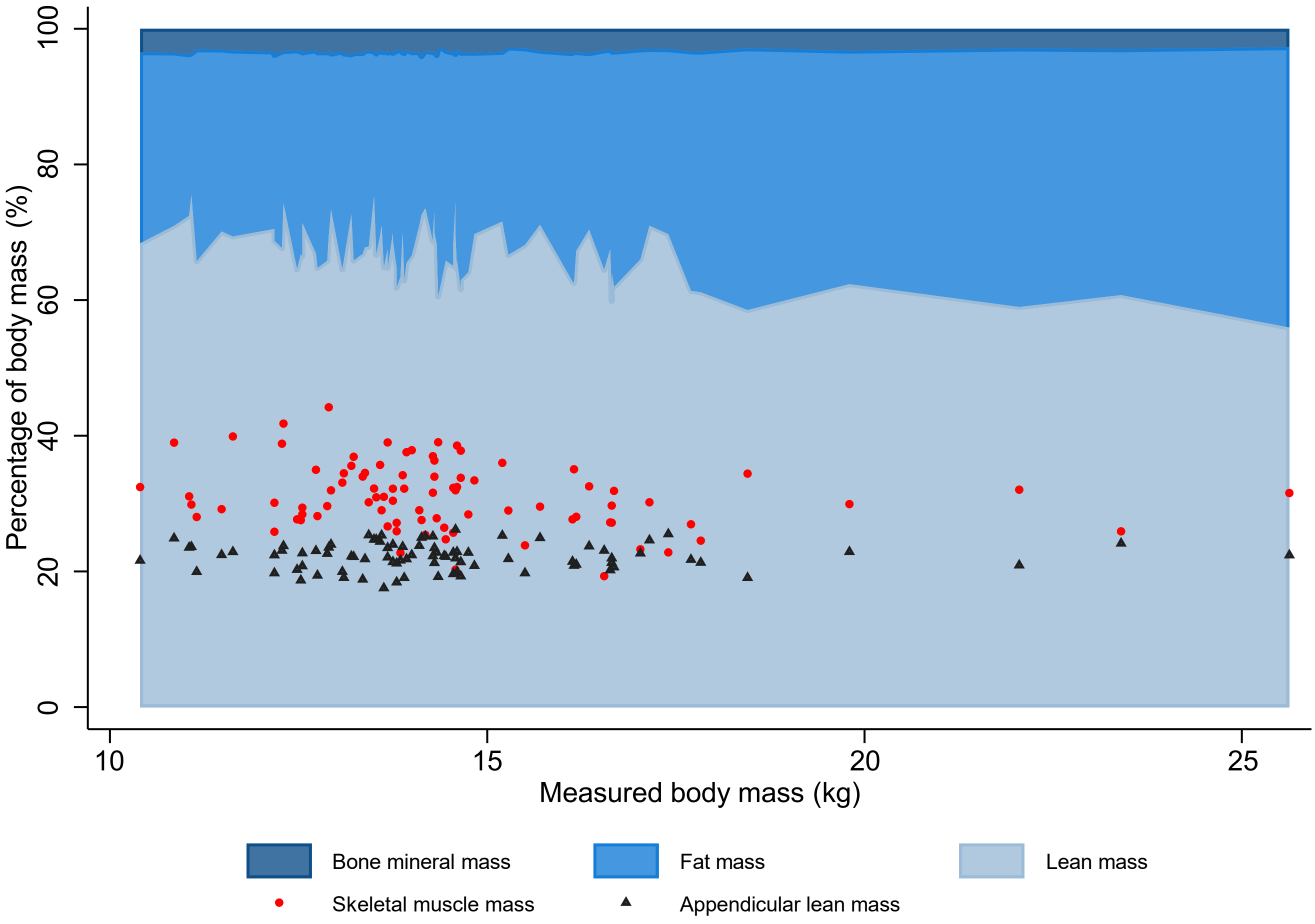

Supplementary Figure 2. Body composition for the BONUSKids+ subsample with complete skeletal muscle mass (SMM) and appendicular lean mass (ALM) data.
Denominator of percentage body mass (y-axis) calculated as the sum of whole-body bone mineral mass, fat mass, and lean mass as measured by dual-energy x-ray absorptiometry. Measured body mass (x-axis) as measured by weighing scale. SMM measured by D_3-creatine dilution method. *N*=87. Bone mineral mass, fat mass, lean mass and ALM measured by dual-energy x-ray absorptiometry.

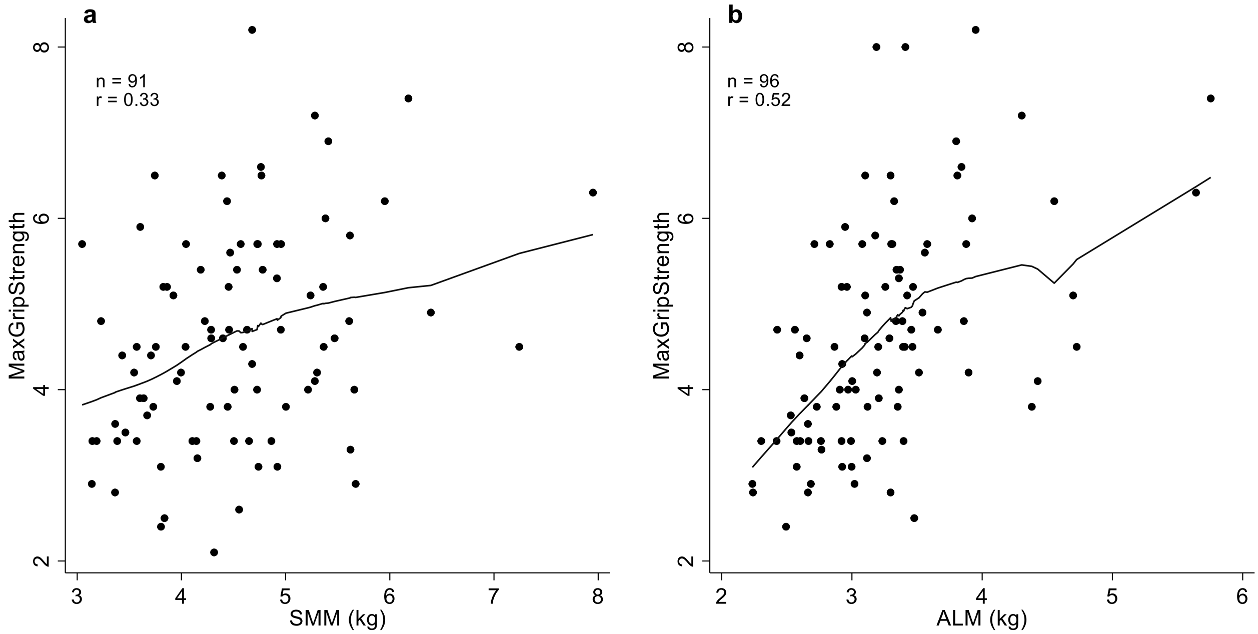

Supplementary Figure 3. Associations of maximum hand-grip strength with skeletal muscle mass (SMM) and appendicular lean mass (ALM).

Maximum hand grip strength is the maximum value of 6 trials. ALM measured by dual-energy x-ray absorptiometry, SMM measured by D_3-creatine dilution method. Lowess line shown with bandwidth of 0.8, correlation shown as Pearson correlation coefficient ($P < 0.01$ for both).

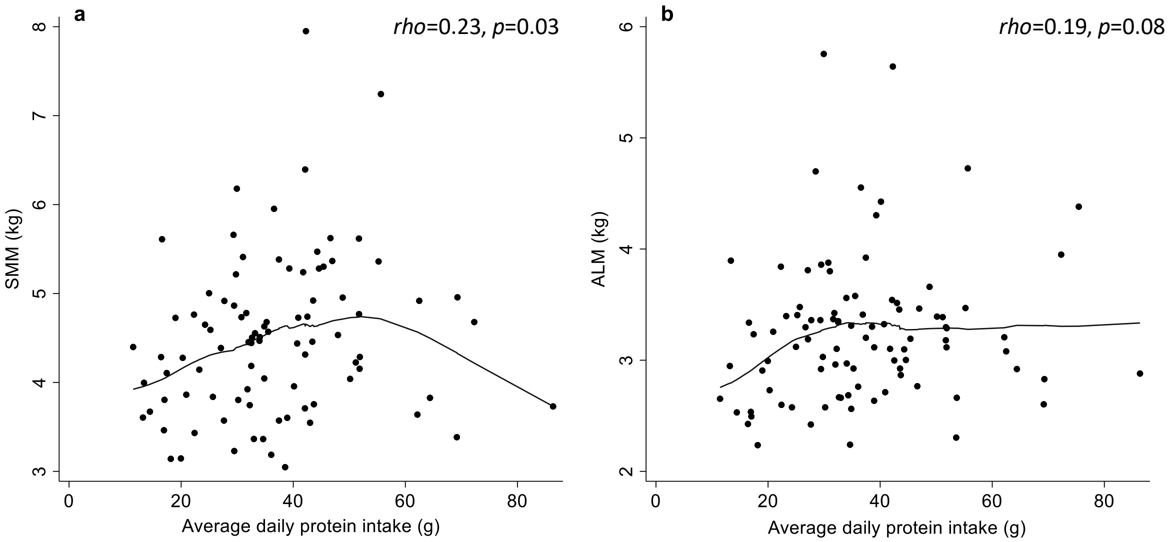

Supplementary Figure 4. Associations of average daily protein intake with skeletal muscle mass (SMM) and appendicular lean mass (ALM).
ALM measured by dual-energy x-ray absorptiometry, SMM measured by D_3-creatine dilution method. Lowess line shown with bandwidth of 0.8. Panel a, n = 90; panel b, n = 95.

Supplementary Table 2. Associations of average hand-grip strength with skeletal muscle mass, appendicular lean mass, and other body composition and anthropometric measures among 4-year-old children in Dhaka, Bangladesh.

Exposure	Pearson correlation			Unadjusted model[d]		Multivariable-adjusted model[e]	
	n	r	P^*	β (95% CI)	P^*	β (95% CI)	P^*
D_3Cr SMM (100g)[a]	91	0.29	0.005	0.034 (0.010, 0.057)	0.005	-0.004 (-0.032, 0.025)	0.8
D_3Cr SMM / total body weight (%)[a]	91	-0.15	0.2	-0.031 (-0.075, 0.013)	0.2	-0.0001 (-0.042, 0.042)	1.0
ALM (100g)[a]	96	0.54	<0.001	0.089 (0.061, 0.118)	<0.001	0.055 (-0.017, 0.128)	0.1
ALM / height^2 (kg/m^2)[b]	96	0.45	<0.001	1.089 (0.646, 1.532)	<0.001	0.763 (-0.011, 1.538)	0.05
ALM /total body weight (%)[a]	96	0.22	0.03	0.122 (0.009, 0.235)	0.03	0.125 (0.018, 0.232)	0.02
Upper limb LM (100g)[a]	96	0.57	<0.001	0.364 (0.256, 0.473)	<0.001	0.282 (0.062, 0.502)	0.01
Lower limb LM (100g)[a]	96	0.51	<0.001	0.112 (0.073, 0.15)	<0.001	0.043 (-0.051, 0.136)	0.4
TBLH LM (100g)[a]	96	0.54	<0.001	0.043 (0.029, 0.057)	<0.001	0.035 (-0.011, 0.081)	0.1
TBLH LM / height^2 (kg/m^2)[b]	96	0.43	<0.001	0.573 (0.325, 0.820)	<0.001	0.425 (-0.057, 0.907)	0.08
BMI-for-age z-score[c]	100	0.38	0.001	0.367 (0.185, 0.548)	<0.001	0.193 (-0.030, 0.416)	0.09
Height (cm)[b]	100	0.49	<0.001	0.121 (0.078, 0.165)	<0.001	0.100 (0.042, 0.157)	0.001
Weight (kg)[c]	100	0.48	<0.001	0.207 (0.130, 0.283)	<0.001	0.102 (-0.059, 0.264)	0.2

ALM appendicular lean mass measured by dual energy X-ray absorptiometry, *LM* lean mass measured by dual X-ray absorptiometry, *SMM* skeletal muscle mass measured by D_3-creatine dilution method, *TBLH* total body less head.

[a] Multivariable model includes child sex, age (months), height (cm), BMI-for-age z-score, maternal height (cm), maternal education, asset index quintile, and maternal MDIG vitamin D supplementation group.

[b] Multivariable model includes child sex, age (months), BMI-for-age z-score, maternal height (cm), maternal education, asset index quintile, and maternal MDIG vitamin D supplementation group

[c] Multivariable model includes child sex, age (months), height (cm), maternal height (cm), maternal education, asset index quintile, and maternal MDIG vitamin D supplementation group.

[d] Estimates are interpreted as the average change in maximum hand-grip strength in kg for every 1-unit change in the body composition or anthropometric variable.

[e] Estimates are interpreted as the average change in maximum hand-grip strength in kg for every 1-unit change in the body composition or anthropometric variable, holding all other covariates constant.

[*] $P < 0.05$ considered statistically significant.

Supplementary Table 3. Associations of maximum hand-grip strength with skeletal muscle mass, appendicular lean mass, and other body composition and anthropometric measures among participants with complete data for skeletal muscle mass, appendicular lean mass and hand-grip strength.

Body composition or anthropometric variable	Pearson correlation			Unadjusted model[d]		Multivariable model[e]	
	n	r	*P**	β (95% CI)	*P**	β (95% CI)	*P**
D_3Cr SMM (100g)[a]	87	0.344	0.001	0.046 (0.019, 0.073)	0.001	0.000003 (-0.032, 0.033)	1.0
D_3Cr SMM / total body weight (%)[a]	87	-0.13	0.2	-0.031 (-0.083, 0.020)	0.2	0.007 (-0.041, 0.055)	0.8
ALM (100g)[a]	87	0.584	<0.001	0.112 (0.079 0.146)	<0.001	0.079 (-0.003, 0.161)	0.06
ALM / height^2 (kg/m^2)[b]	87	0.47	<0.001	1.293 (0.766, 1.821)	<0.001	0.885 (-0.021, 1.791)	0.06
ALM / total body weight (%)[a]	87	0.22	0.04	0.140 (0.007, 0.273)	0.04	0.153 (0.031, 0.274)	0.01
Upper limb LM (100g)[a]	87	0.608	<0.001	0.439 (0.315 0.563)	<0.001	0.333 (0.088, 0.579)	0.01
Lower limb LM (100g)[a]	87	0.562	<0.001	0.144 (0.098 0.189)	<0.001	0.078 (-0.032, 0.188)	0.2
TBLH LM (100g)[a]	87	0.584	<0.001	0.054 (0.038 0.07)	<0.001	0.047 (-0.004, 0.098)	0.07
TBLH LM / height^2 (kg/m^2)[b]	87	0.44	<0.001	0.672 (0.377, 0.967)	<0.001	0.437 (-0.121, 0.996)	0.1
BMI-for-age z-score[c]	87	0.422	<0.001	0.458 (0.246 0.671)	<0.001	0.162 (-0.090, 0.414)	0.2
Height (cm)[b]	87	0.543	<0.001	0.152 (0.101 0.203)	<0.001	0.128 (0.061, 0.195)	<0.001
Weight (kg)[c]	87	0.539	<0.001	0.259 (0.172 0.347)	<0.001	0.079 (-0.101, 0.259)	0.4

ALM appendicular lean mass measured by dual energy X-ray absorptiometry, *LM* lean mass measured by dual X-ray absorptiometry, *SMM* skeletal muscle mass measured by D_3-creatine dilution method, *TBLH* total body less head.

[a] Multivariable model includes child sex, age (months), height (cm), BMI-for-age z-score, maternal height (cm), maternal education, asset index quintile, and maternal MDIG vitamin D supplementation group.

[b] Multivariable model includes child sex, age (months), BMI-for-age z-score, maternal height (cm), maternal education, asset index quintile, and maternal MDIG vitamin D supplementation group.

[c] Multivariable model includes child sex, age (months), height (cm), maternal height (cm), maternal education, asset index quintile, and maternal MDIG vitamin D supplementation group.

[d] Estimates are interpreted as the average change in maximum hand-grip strength in kg for every 1-unit change in the body composition or anthropometric variable.

[e] Estimates are interpreted as the average change in maximum hand-grip strength in kg for every 1-unit change in the body composition or anthropometric variable, holding all other covariates constant.

[*] $P < 0.05$ considered statistically significant

Supplementary Table 4. Associations of maximum hand-grip strength with skeletal muscle mass, appendicular lean mass, and other body composition and anthropometric measures, stratified by sex, among 4-year-old children in Dhaka, Bangladesh.

Models	Pearson correlation			Unadjusted model		Multivariable adjusted model	
	n	r	P^*	β (95% CI)[d]	P^*	β (95% CI)[e]	P^*
D_3Cr SMM (100g)[a]							
Boys	47	0.39	0.007	0.053 (0.015, 0.090)	0.007	0.015 (-0.040, 0.071)	0.6
Girls	44	0.23	0.1	0.032 (-0.011, 0.074)	0.1	-0.012 (-0.062, 0.038)	0.6
ALM (100g)[a]							
Boys	49	0.53	<0.001	0.094 (0.050, 0.138)	<0.001	0.023 (-0.102, 0.148)	0.7
Girls	47	0.56	<0.001	0.181 (0.101, 0.261)	<0.001	0.194 (0.055, 0.332)	0.008
Upper limb LM (100g)[a]							
Boys	49	0.63	<0.001	0.423 (0.27, 0.577)	<0.001	0.359 (0.018, 0.701)	0.04
Girls	47	0.48	<0.001	0.652 (0.289, 1.014)	<0.001	0.519 (-0.038, 1.076)	0.07
Lower limb LM (100g)[a]							
Boys	49	0.49	<0.001	0.112 (0.053, 0.172)	<0.001	-0.032 (-0.192, 0.127)	0.7
Girls	47	0.57	<0.001	0.227 (0.128, 0.326)	<0.001	0.247 (0.074, 0.420)	0.006
TBLH LM (100g)[a]							
Boys	49	0.56	<0.001	0.050 (0.028, 0.071)	<0.001	0.065 (-0.005, 0.135)	0.07
Girls	47	0.48	<0.001	0.072 (0.032, 0.111)	<0.001	0.040 (-0.067, 0.146)	0.5
BMI-for-age z-score[b]							
Boys	52	0.41	0.003	0.451 (0.163, 0.740)	0.003	0.306 (-0.076, 0.688)	0.1
Girls	48	0.32	0.03	0.437 (0.048, 0.826)	0.03	0.295 (-0.178, 0.769)	0.2
Height (cm)[c]							
Boys	52	0.45	<0.001	0.124 (0.054, 0.194)	<0.01	0.076 (-0.016, 0.168)	0.1
Girls	48	0.47	<0.001	0.185 (0.082, 0.288)	<0.001	0.203 (0.048, 0.358)	0.01
Weight (kg)[b]							
Boys	52	0.48	<0.001	0.223 (0.106, 0.339)	<0.001	0.182 (-0.086, 0.451)	0.2
Girls	48	0.46	<0.001	0.347 (0.150, 0.544)	<0.001	0.216 (-0.155, 0.586)	0.2

ALM appendicular lean mass measured by dual energy X-ray absorptiometry, *LM* lean mass measured by dual X-ray absorptiometry, *SMM* skeletal muscle mass measured by D_3-creatine dilution method, *TBLH* total body less head.

[a] Multivariable model includes child sex, age (months), height (cm), BMI-for-age z-score, maternal height (cm), maternal education, asset index quintile, and maternal MDIG vitamin D supplementation group.

[b] Multivariable model includes child sex, age (months), height (cm), maternal height (cm), maternal education, asset index quintile, and maternal MDIG vitamin D supplementation group.

[c] Multivariable model includes child sex, age (months), BMI-for-age z-score, maternal height (cm), maternal education, asset index quintile, and maternal MDIG vitamin D supplementation group.

[d] Estimates are interpreted as the average change in maximum hand-grip strength in kg for every 1-unit change in the body composition or anthropometric variable.

[e] Estimates are interpreted as the average change in maximum hand-grip strength in kg for every 1-unit change in the body composition or anthropometric variable, holding all other covariates constant.

[*] $P < 0.05$ considered statistically significant.

References

1 Shaheen N, R. A., Mohiduzzaman M, Parvin Banu C, Bari M, Basak Tukun A. Food Composition Table for Bangladesh. *Institute of Nutrition and Food Science, Centre for Advanced Research in Sciences, University of Dhaka* (2013).

2 Rhee, J. J. et al. Comparison of methods to account for implausible reporting of energy intake in epidemiologic studies. *Am J Epidemiol* **181**, 225-233 (2015).
